# Revisiting Exclusion of Prior Cancer in Clinical Trials of Male Breast Cancer

**DOI:** 10.7150/jca.80740

**Published:** 2023-03-21

**Authors:** Aniruddha Rathod, Caitlin C. Murphy, Asal Rahimi, Sandi L. Pruitt

**Affiliations:** 1 Peter O'Donnell Jr School of Public Health, University of Texas Southwestern Medical Center, Dallas TX; 2 Department of Health Promotion and Behavioral Sciences, School of Public Health, University of Texas Health Science Center at Houston, Houston, TX; 3 Department of Radiation Oncology, University of Texas Southwestern Medical Center, Dallas, TX; 4 Harold C. Simmons Comprehensive Cancer Center, University of Texas Southwestern Medical Center, Dallas TX

**Keywords:** Male breast cancer, SEER, prior cancer, multiple primary malignancy, exclusion criteria, clinical trials

## Abstract

**Background:** Eligibility criteria for cancer clinical trials present challenges to enrollment. Many trials exclude patients with a prior cancer. This common practice may be especially detrimental to trials of rare cancers, such as male breast cancer, that struggle to accrue adequate numbers of participants.

**Objectives:** To estimate prevalence of prior cancer among men newly diagnosed with breast cancer and describe characteristics of men with prior cancer compared to those without.

**Methods:** We identified men diagnosed with breast cancer between 2011-2015 using population-based data from National Cancer Institute's Surveillance, Epidemiology, and End Results program of cancer registries. We used sequence number and diagnosis year to identify cancers diagnosed prior to breast cancer (inclusive of prior breast, different, and unknown types of cancer). We compared sociodemographic, tumor, and treatment characteristics of men with and without prior cancer using chi-square tests.

**Results:** Among 2317 men, nearly one quarter (24.3%) had any prior cancer, and the majority (58.7%) of these were of a different cancer type. A higher proportion of men with a prior cancer of a different type were older, had smaller (≤ 2 cm) breast tumors, were diagnosed with stage 0-1 breast cancer, and did not receive surgery compared to men without any prior cancer; there were no statistically significant differences by race and ethnicity, county median income, hormone receptor status, or surgery type.

**Conclusion:** Given prevalence of prior cancer in this rare and understudied population of men diagnosed with breast cancer, including men with prior cancer in clinical trials may improve accrual.

## Introduction

As of 2019, 67% of cancer survivors had lived more than five years since their cancer diagnosis, 47% more than 10 years, and 18% more than 20 years. The number of U.S. cancer survivors is projected to grow to 26.1 million by 2040 1. During the time period 2009—2013, nearly 20% of cancer survivors in the US were affected by more than one cancer, 2 and this proportion is expected to increase in the future.

Cancer survivors are commonly excluded from cancer clinical trials. This has substantial impact on study accrual and may limit generalizability of trial findings. For example, our previous study demonstrated that 18% of patients were ineligible for lung cancer clinical trials due to prior cancer being an exclusion criterion 3. Such exclusion criteria may be especially detrimental for trials of rare cancers, such as male breast cancer. In the U.S., an estimated 2710 men will be diagnosed with breast cancer in 2022 4. Prior cancer was an exclusion criterion in 66% of breast cancer clinical trials with 47% of trials stipulating no prior cancer within 5 years of the breast cancer diagnosis 5. A study examining the inclusion and representation of men in breast cancer trials between January 1, 2000 and April 31, 2017 failed to identify a single trial that had successfully completed accrual for male breast cancer, and most breast cancer clinical trials excluded men 6. Another study identified 131 phase III trials for therapeutic interventions, and less than 1% of participants were men 7. As of August 2022, there were only eight actively recruiting trials specifically for male breast cancer in the world, 8 and consequently, findings from female breast cancer trials are used to inform treatment of male breast cancer. This is especially concerning because, given increasing use of targeted therapies and the differences in male and female breast cancer biology 9, 10, results from trials comprised of women may not be generalizable to men. Given that men with breast cancer, compared to women, have 19% higher overall mortality 11, it is clear that clinical trials in this population are sorely needed. To assess the potential impact of excluding patients with prior cancer from clinical trials, we estimated the prevalence and characteristics of prior cancer among men newly diagnosed with breast cancer. Findings will inform the impact of prior cancer on clinical trial accrual.

## Methods

We used population-based data from National Cancer Institute's Surveillance, Epidemiology, and End Results (SEER) program of cancer registries 12 in the study. We included adult (age ≥18 years) men diagnosed with breast cancer during 2011-2015. SEER 17 includes the following registries: Alaska Native Tumor Registry, Connecticut, Detroit, Atlanta, Greater Georgia, Rural Georgia, San Francisco-Oakland, San Jose-Monterey, Greater California, Hawaii, Iowa, Kentucky, Los Angeles, Louisiana, New Mexico, New Jersey, Seattle-Puget Sound, and Utah. For men with more than one breast cancer diagnosed in 2011-2015 (n=31), the most recent breast cancer diagnosis was selected as the index breast cancer. Prior cancer was defined as having any primary cancer prior to diagnosis of the index breast cancer, and we used the SEER variable *sequence number,* i.e., order of all primary tumors, and diagnosis year to identify prior cancers. Men with only one primary (index) breast cancer were identified as not having any prior cancer, and those with one or multiple prior primary cancers (i.e., one or more cancers before index breast cancer) were identified as having any prior cancer. We estimated prevalence of prior cancer of a different type (i.e., type other than breast cancer) and described characteristics of men with and without a prior cancer of a different type, including age at breast cancer diagnosis, race and ethnicity, median household income at the county level, breast cancer stage (AJCC 7^th^ edition), tumor size (in centimeters), hormone receptor type, receipt of surgery, chemotherapy, and radiation (yes/no), and type of surgery (mastectomy/ breast conserving surgery). We compared these characteristics using chi-square or Fisher's exact tests.

## Results

We identified 2348 incident breast cancers diagnosed during 2011-2015 among 2317 men, of whom nearly one quarter (n=564, 24.3%) had any prior cancer. As illustrated in Figure S1, among those who had any prior cancer (n=564), 331 men had prior cancer of a different type, 55 men had prior breast cancer, and 178 men had prior cancer of unknown type. The prevalence of prior cancer of a different type varied by age; 4% among men less than 65 years of age and 10.3% among men 65 years of age or older. Among men with prior cancer of a different type (n=331), 60% of these cancers were diagnosed ≤ 5 years before the index breast cancer. The most common types of prior cancers of a different type (n=331) included prostate (40.2%), urinary bladder (6%), lung and bronchus (5.7%), and skin melanoma (5.1%).

**Table 1** summarizes characteristics of men with (n=331) a prior cancer of a different type and men without any prior cancer (n=1753). Compared to men without any prior cancer, a higher proportion of men with prior cancer of a different type were older, i.e., ≥ 75 years, diagnosed with stage 0-I breast cancer, had smaller (≤ 2 cm) breast tumors, and did not receive surgery. We did not observe any statistically significant differences between the two groups by race and ethnicity, median household income, hormone receptor type, or type of surgery. **Table S1** summarizes characteristics of men with a prior breast cancer (n=55) and men without any prior cancer (n=1753).

## Discussion

In this study, we used US population-based data from the SEER program of cancer registries to estimate prevalence and describe characteristics of prior cancer among men newly diagnosed with breast cancer. Nearly a quarter (24%) of men had any prior cancer. Notably, (Table 1) most men with prior cancer were previously diagnosed with a different type of cancer, fewer had a prior breast cancer. Prior cancer prevalence is rising and is anticipated to continue to increase due to early detection, advanced therapies 13, 14, and an aging population.

The practice of excluding individuals with prior cancer from clinical trials limits trial accrual and generalizability of findings, and it substantially affects trials of rare cancers diagnosed among older adults where accrual is difficult. We demonstrated that this exclusion is especially impactful for men with breast cancer, given the substantial prevalence (24%) of any prior cancer. Most cancer clinical trials exclude persons with a prior cancer diagnosed within five years of the index cancer 15, and findings from this study demonstrated that 60% of men with prior cancer of a different type were diagnosed within this window, thereby excluding a substantial proportion of patients. Individuals with prior cancer are excluded due to their perceived risk of not being able to tolerate the treatment or previous treatment interfering with the trial outcomes. However, this assumption is not evidence based—a recent study among women with breast cancer showed prior cancer did not adversely impact overall survival 16. However, such a study has not been performed for male breast cancer. Future research is needed to assess whether prior cancer impacts survival among men with breast cancer and additional research is needed about prior cancer prevalence, particularly for other rare cancers.

Clinical trial accrual is challenging given that male breast cancer is a rare cancer. We are among the first to determine prior cancer prevalence in this population and demonstrate that many characteristics differ for men with and without a prior cancer of a different type. Therefore, our findings are novel and suggest that including men with prior cancer in breast cancer clinical trials may improve accrual and generalizability of findings. Previous studies have examined prevalence of prior cancer of other cancer types. For example, a population-based study found the prevalence of prior cancer among individuals newly diagnosed with cancer during 2009-2013 ranged from 4-37%, depending on cancer type 2. Notably, among women with breast cancer, prevalence of prior cancer of a different type was 3.7% or 7.4%, for women less than 65 years of age and women 65 years of age or older, respectively. In contrast, our present study among men illustrates that prevalence of prior cancer of a different type was somewhat higher, at 4% or 10.3%, for men less than 65 years of age and men 65 years of age or older, respectively.

One of the limitations of our study is that the type of prior cancer was unknown for 31% of men with any prior cancer. Nevertheless, these findings highlight the importance of considering prior cancer when designing clinical trials for rare cancers. As prior cancer was common in this understudied population of men diagnosed with breast cancer, including patients with prior cancer in clinical trials may improve accrual and generalizability of findings.

## Supplementary Material

Supplementary figure and table.

## Figures and Tables

**Table 1:** Characteristics of men diagnosed with breast cancer 2011-2015, for those with prior cancer of a different type and those without any prior cancer (n=2084).

Characteristics	No prior cancer (n=1753) n (%)	Prior cancer of a different type (n=331) n (%)	p-Value
**Age at breast cancer diagnosis (in years)**			<0.01
≤ 50	324 (18.5)	39 (11.8)	
> 50 to ≤65	653 (37.3)	63 (19)	
>65 to ≤75	486 (27.7)	109 (32.9)	
≥ 75	290 (16.5)	120 (36.3)	
**Race and ethnicity**			0.15
Non-Hispanic White	1266 (72.2)	256 (77.3)	
Non-Hispanic Black	236 (13.5)	40 (12.1)	
Hispanic White	138 (7.9)	17 (5.1)	
Other	100 (5.7)	18 (5.4)	
Unknown	13 (0.7)	0	
**Median household income (in thousands) at county level (in USD)**			0.69
<40	100 (5.7)	14 (4.2)	
40 to <55	305 (17.4)	57 (17.2)	
55 to <70	701 (40)	131 (39.6)	
≥ 70	646 (36.9)	129 (39)	
Missing	1		
**Breast cancer stage (AJCC 7^th^ edition)**			<0.01
0-I	531 (31.8)	131 (42.5)	
II	713 (42.8)	117 (38)	
III	277 (16.6)	34 (11)	
IV	146 (8.8)	26 (8.5)	
Missing	86	23	
**Tumor size (in cm)**			<0.01
≤ 2	730 (44.5)	164 (53.8)	
>2 to ≤ 5	799 (48.7)	133 (43.6)	
>5 to ≤20	111 (6.8)	8 (2.6)	
Missing	113	26	
**Subtype**			0.98
Luminal A	1324 (85.8)	239 (85.7)	
Luminal B	171 (11.1)	30 (10.8)	
HER-2 enriched	15 (1)	3 (1)	
Triple negative	33 (2.1)	7 (2.5)	
Missing	210	52	
**Surgery receipt**			<0.01
Yes	1534 (87.5)	279 (84.3)	
No	208 (11.9)	44 (13.3)	
Unknown	11 (0.6)	8 (2.4)	
**Type of Surgery**			0.62^f^
Mastectomy	1349 (87.9)	242 (86.7)	
Breast conserving surgery	184 (12)	37 (13.3)	
Unknown	1 (0.1)	0	
Missing	219	52	
**Chemotherapy**			<0.01
Yes	679 (38.7)	73 (22.1)	
No/Unknown	1074 (61.3)	258 (77.9)	
**Radiation**			<0.01
Yes	483 (27.6)	65 (19.6)	
No/Unknown/Refused	1270 (72.4)	266 (80.4)	

f - Fischer's exact test p-value. Note: cases with missing data were excluded from chi-square and Fischer's exact tests.
