# Supplementary material for: Revisiting Exclusion of Prior Cancer in Clinical Trials of Male Breast Cancer
Source: J Cancer. 2023 Mar 21;14(5):737–40. doi: 10.7150/jca.80740 (PMC10088891; doi:10.7150/jca.80740)

Supplement Material

Table S1: Characteristics of men diagnosed with breast cancer 2011-2015, for those with prior breast cancer and those without any prior cancer (n=1,808).

Characteristics	No prior cancer (n=1753) n (%)	With prior breast cancer (n=55) n (%)	p-Value
**Age at breast cancer diagnosis (in years)**			0.32
≤ 50	324 (18.5)	8 (14.6)	
> 50 to ≤65	653 (37.3)	16 (29.1)	
>65 to ≤75	486 (27.7)	18 (32.7)	
≥ 75	290 (16.5)	13 (23.6)	
**Race and ethnicity**			0.1[f]
Non-Hispanic White	1266 (72.2)	36 (65.5)	
Non-Hispanic Black	236 (13.5)	8 (14.6)	
Hispanic White	138 (7.9)	10 (18.2)	
Other	100 (5.7)	1 (1)	
Unknown	13 (0.7)	0	
**Median household income (in thousands) at county level (in USD)**			0.31
<40	100 (5.7)	3 (5.6)	
40 to <55	305 (17.4)	15 (27.3)	
55 to <70	701 (40)	19 (34.6)	
≥ 70	646 (36.9)	18 (32.7)	
Missing	1		
**Breast cancer stage (AJCC 7th edition)**			<0.01
0-I	531 (31.8)	29 (60.4)	
II	713 (42.8)	7 (14.6)	
III	277 (16.6)	5 (10.4)	
IV	146 (8.8)	7 (14.6)	
Missing	86	7	
**Tumor size (in cm)**			<0.01
≤ 2	730 (44.5)	36 (76.6)	
>2 to ≤ 5	799 (48.7)	9 (19.1)	
>5 to ≤20	111 (6.8)	2 (4.3)	
Missing	113	8	
**Subtype**			0.43[f]
Luminal A	1324 (85.8)	43 (89.6)	
Luminal B	171 (11.1)	3 (6.3)	
HER-2 enriched	15 (1)	1 (2.1)	
Triple negative	33 (2.1)	1 (3)	
Missing	210	7	
**Surgery receipt**			0.31
Yes	1534 (87.5)	45 (81.8)	
No	208 (11.9)	10 (18.2)	

Unknown	11 (0.6)	0 (0)	
**Type of Surgery**			0.65[f]
Mastectomy	1349 (87.9)	41 (91.1)	
Breast conserving surgery	184 (12)	4 (8.9)	
Unknown	1 (0.1)	0	
Missing	219	10	
**Chemotherapy**			0.53
Yes	679 (38.7)	19 (34.5)	
No/Unknown	1074 (61.3)	36 (65.5)	
**Radiation**			0.52
Yes	483 (27.6)	13 (23.6)	
No/Unknown/Refused	1270 (72.4)	42 (76.4)	

f - Fischer's exact test p-value. Note: cases with missing data were excluded from chi-square and Fischer's exact tests.

Figure S1: Study flowchart illustrating the patterns of prior cancer history

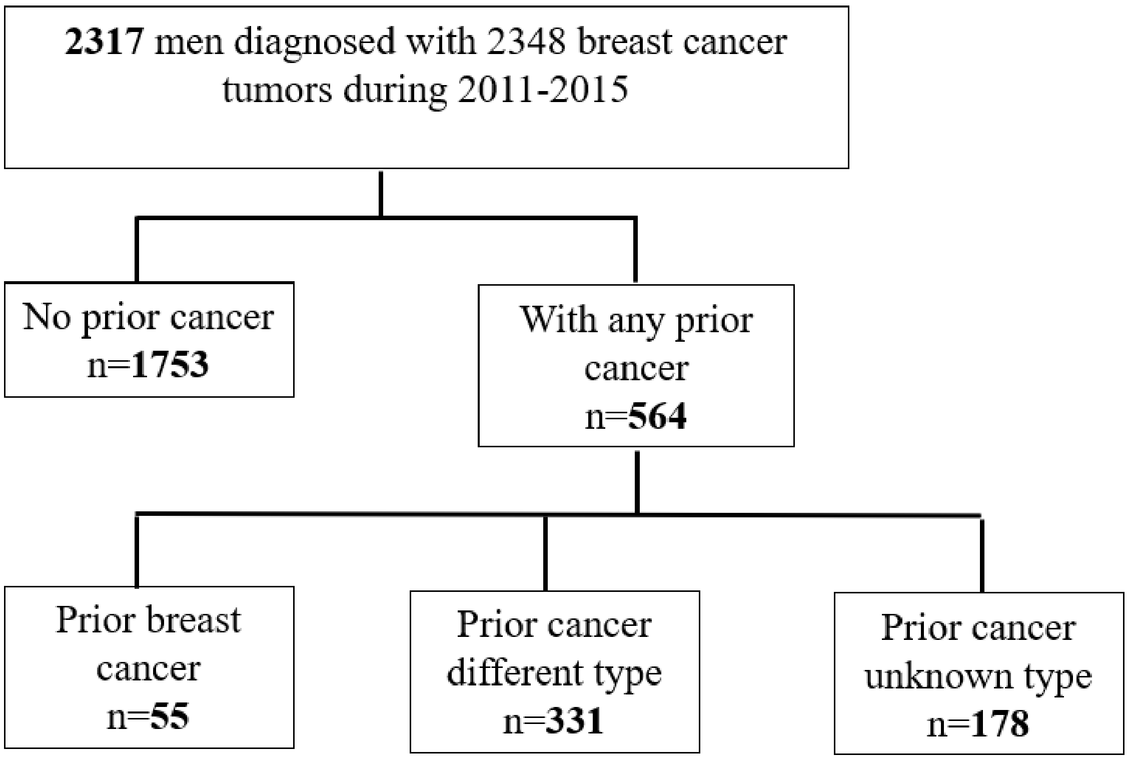

Supplement: Supplementary file 1 — Supplementary figure and table. [file jcav14p0737s1.pdf]
